# Characterizing and configuring motorized wedge for a new generation telecobalt machine in a treatment planning system

**DOI:** 10.4103/0971-6203.31147

**Published:** 2007

**Authors:** Rajesh A. Kinhikar, Smriti Sharma, Rituraj Upreti, Chandrashekhar M. Tambe, Deepak D. Deshpande

**Affiliations:** Department of Medical Physics, Tata Memorial Hospital, Parel, Mumbai - 400012, India

**Keywords:** Motorized wedge, quality assurance, telecobalt, treatment planning

## Abstract

A new generation telecobalt unit, Theratron Equinox-80, (MDS Nordion, Canada) has been evaluated. It is equipped with a single 60-degree motorized wedge (MW), four universal wedges (UW) for 15°, 30°, 45° and 60°. MW was configured in Eclipse (Varian, Palo Alto, USA) 3D treatment planning system (TPS). The profiles and central axis depth doses (CADD) were measured with radiation field analyzer blue water phantom for MW. These profiles and CADD for MW were compared with UW in a homogeneous phantom generated in Eclipse for various field sizes. The absolute dose was measured for a field size of 10 × 10 cm2 only in a MEDTEC water phantom at 10 cm depth with a 0.13 cc thimble ion chamber (Scanditronix Wellhofer, Uppsala, Sweden) and a NE electrometer (Nuclear Enterprises, UK). Measured dose with ion chamber was compared with the TPS predicted dose. MW angle was verified on the Equinox for four angles (15°, 30°, 45° and 60°). The variation in measured and calculated dose at 10 cm depth was within 2%. The measured and the calculated wedge angles were in well agreement within 2°. The motorized wedges were successfully configured in Eclipse for four wedge angles.

## Introduction

In the era of advanced radiotherapy (RT) technology development, the telecobalt units (TCU) are still trying to compete with the linear accelerator (LINAC). Physical wedges have been used for many decades. Further developments in the head design of the TCU have brought the concept of “universal wedges (UW) and motorized wedge (MW)”. MW is a single physical wedge (60°) which could generate desired angle (0 to 60°) with the combination of open and wedged beam.[1,2] In our department, we have Eclipse, Version 7.3 system (Varian, Palo Alto, USA) 3 dimensional treatment planning system (3DTPS). With the help of measured central axis depth doses (CADD), wedge profiles for 60° MW and adjusting beam weight, the isodoses can be tilted to get the desired wedge angle.

Recently, coping with all these new technology, a new generation TCU, Theratron Equinox-80, (MDS Nordion, Canada) has been first installed at Tata Memorial Hospital in June 2006. Before clinical introduction of MW in the department, it was required to verify the performance of the treatment planning[3,4] process with MW. Since the treatment time for the arbitrary MW angle cannot be predicted manually, it was needed to characterize and configure MW in eclipse. The objective of this paper was to report on the configuration of MW in eclipse and discuss the results.

## Materials and Methods

### Design of MW

The MW provides a 60° nominal wedge angle. The maximum field size covered by the MW is 15 × 20 cm^2^. Alternately, it can be moved in and out of the radiation fields. It consists of a 60-degree wedge mounted in the asymmetric collimator below the lead leaves and above the tungsten trimmer bars. For convenience of access, the MW and UW filters are inserted transversely. When the collimator is at zero angle position, the MW is oriented with the thin edge directed to the left when facing the gantry and the UW is oriented with the thin edge directed to the right when facing the gantry.

Basically the concept of MW is not new; however, it is a new concept in cobalt units. On the graphical user interface control console of the Equinox-80, the treatment time is set for total open beam time and the motorized wedge beam time. In Equniox-80 machine, the desired MW angle is achieved as follows:

- The source is exposed for the open beam time
- The source is retracted
- The MW goes to the In position
- The source moves to the fully exposed position
- The source remains in the fully exposed position for the wedged time
- At the end of the wedged time, the source is retracted
- The MW goes to the out position and the treatment is complete.

In eclipse, motorized wedge with 60° was commissioned with the measured CADD and the profile data. The dose distribution of fields containing a MW is calculated on the basis of the weighted dose percentages for the wedged and open parts of the field. The Eclipse then displays the monitor unit (MU) values and reference dose for both the open and wedged part of a field containing a MW.

The weight factor is a parameter used for MW to indicate the dose percentage of the wedged and open part of a field containing a wedge. The weight factors range from 0 to 1.0 and the factor indicates the following:

0 - fully open field,

1 - fully wedged field

Other values - partly wedged field.

For example, a weight factor of 0.5 for a 60° MW is approximately equivalent to a field containing a 30° UW or a standard wedge.

The dose box in eclipse shows the coefficient used to indicate the MUs of the wedged and open part of the field. The weight factor box shows the weight of the wedged part of the field. The coefficient indicating the MUs of the wedged and open part of the field is calculated as follows:

(1) $$\text{D}={\text{MU}}_{\text{wedge}}/{\text{MU}}_{\text{Total}},$$

Where, ${\text{MU}}_{\text{Total}}={\text{MU}}_{\text{open}}+{\text{MU}}_{\text{wedge}}$

By using the appropriate beam weights, it is possible to generate isodose distributions, which exhibit effective wedge angles ranging from 0 to 60°. The universal wedge equation may be used to determine the beam weights. However, the beam weight factor may differ from the TPS to TPS based on the algorithm and the calculation module used. This universal wedge equation relates the beam weight of the wedged field to the effective wedge angle produced by combining the wedged field to the non-wedged field and is expressed as

(2) tan (theta)=B. tan (theta W)

Where, B is the normalized weight imposed on wedged field, theta w is the maximum wedge angle of the wedge filter and theta is the effective wedge angle. Thus the equation (2) can also be written as:

(3) wedges beam weight={ tan (desired angle)/tan (motorized wedge angle) }

In eclipse, the 3D dose distribution in a homogeneous phantom is calculated using the selected calculation model. The dose distributions are calculated for the field sizes selected from among the original set of CADD and profile measurements.

### Measurements of beam parameters

**Figure 1:**
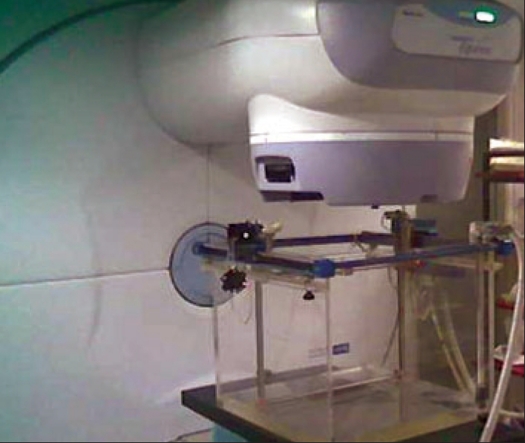
Shows the setup for beam data generation using blue water phantom for Equinox-80. CADD were measured for 30 cm depth for square field sizes (2×2 cm^2^ to 15×15 cm^2^) and a maximum-wedged rectangular field size (15 × 20 cm^2^). Off-axis cross-plane profiles were measured for same field sizes at five depths (0.5, 5, 10, 20 and 30 cm).

The data for 60° MW was generated as per the requirement of eclipse TPS. Measurements were carried out using 48 × 48 × 48 cm blue water phantom (Wellhofer Dosimetrie, Schwarzenbruck, Germany) utilizing CU500E Electrometer, a CC13 (0.13 cc) thimble ionization chamber (Scanditronix Wellhofer, Uppsala, Sweden). Figure 1 shows the setup for beam data generation using blue water phantom for Equinox-80. CADD were measured up to 30 cm depth for square field sizes (2×2 cm^2^ to 15×15 cm^2^) and a maximum wedged rectangular field size (15 × 20 cm^2^). Off-axis cross-plane profiles were measured for same field sizes at five depths (0.5, 5, 10, 20 and 30 cm). An in-plane (longitudinal) profile was also measured for maximum wedged field size (15 × 20 cm^2^) at five depths as mentioned above. Measured CADD and profiles were then compared with the data supplied by the manufacturer. The 60° MW and UW factor was measured for field sizes (5, 10, 15 and 15×20 cm^2^) at 10 cm depth in a 30 × 30 × 30 cm^3^ MEDTEC water phantom. A CC13 ion chamber (Scanditronix Wellhofer, Uppsala, Sweden) and the NE electrometer (Nuclear Enterprises, UK) were used for this purpose. The MW factor was compared with UW.

### Calculation of beam parameters

A hybrid homogeneous water phantom (30 × 30 × 30 cm^3^) was generated in Eclipse and was used for commissioning purpose. Two beams of 10 × 10 cm^2^ with a universal 60° and a motorized 60° each were placed at source to surface distance of 80 cm. A dose of 2 Gy was prescribed at the 10 cm depth. The dose calculation was carried out with pencil beam algorithm with a grid size of 2.5 mm. The calculated CADD and the dose profiles for 60° MW beam were then compared with that of the UW beam for field sizes (5, 10, 15 and 15 × 20 cm^2^). The desired (15°, 30° and 45°) MW angle was obtained with optimizing the weight of the motorized wedge beam. The planned (calculated) dose was compared with the measured dose for the above MW angles at 10 cm depth in phantom.

### Verification of dose delivery and wedge angles

#### Absolute dose verification

For the particular MW angle and the respective open and MW beam weights, the dose was measured for a field size of 10 × 10 cm^2^ only in a MEDTEC water phantom at 10 cm depth with a 0.13 cc thimble ion chamber and a NE electrometer. The ion chamber and the electrometer was calibrated for absorbed dose to water (N_dw_) at the National Standard Laboratory, Bhabha Atomic Research Centre (BARC, Mumbai, India). For dosimetry, TRS 398 protocol from IAEA was used. Measured dose with ion chamber was compared with the TPS calculated dose.

#### MW angle verification

An ion chamber array (CA24, Scanditronix Wellhofer, Sweden) with MDS-240 data acquisition system was used for wedge CADD and off-axis profiles for three MW angles (15, 30 and 45°). CA24 contains 24 ion chambers (0.13 cc). The central element provides the relative dose on the central axis. The calibration factor for all the 23 detectors are stored in Canbus Module connected to MDS-240 system, for correction of individual variation in dose response. The chambers are spaced 8 mm apart from each other. The system is able to obtain resolutions of 10, 5, 2.5 and 1.25 mm by shifting the CA24 array and acquiring charge accumulations for repeated irradiations of the same wedge. A 2.5 mm resolution was used in all profiles. The motorized wedge treatment time calculated by eclipse for a particular wedge angle was set on equinox and profiles for 10 × 10 cm^2^ field size were measured at 4 depths (0.5, 5, 10 and 15 cm). The isodoses were generated from the measured data to get the measured MW angles, which then were compared with the calculated MW angle with eclipse.

## Results

Measured CADD with MW for 5, 10, 15 and 15 × 20 cm^2^ field sizes was found in well agreement with CADD supplied by manufacturer within 1% for depths ranging from 0 to 30 cm. Similarly, the profiles were also compared and found within 1% variation. Table 1 shows the comparison of motorized and universal 60°-wedge factor for 5, 10, 15 and 15 × 20 cm^2^ field sizes. From 5 × 5 cm field size to 15 × 15 cm filed size, the wedge factors were found to be constant with the field size. Maximum variation of 0.8% was observed between MWF and UWF for 5 × 5 cm^2^ field size.

**Table 1:** The comparison of motorized and universal 60°-wedge factor for 5, 10, 15 and 15 × 20 cm^2^ field sizes. The wedge factor was measured at 10 cm depth

*Field size (cm^2^)*	*WF (quoted by manufacturer)*	*MWF (60 degree)*	*UWF (60 degree)*	*% variation (MW vs UW)*
5	0.251	0.252	0.254	0.79
10	0.259	0.26	0.262	0.76
15	0.27	0.270	0.272	0.74
15×20	0.273	0.271	0.272	0.37

**Figure 2:**
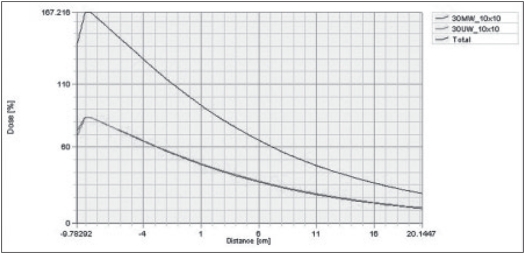
Shows the comparison of calculated CADD of MW and UW for 30° for 10 × 10 cm^2^ field size.

**Figure 3:**
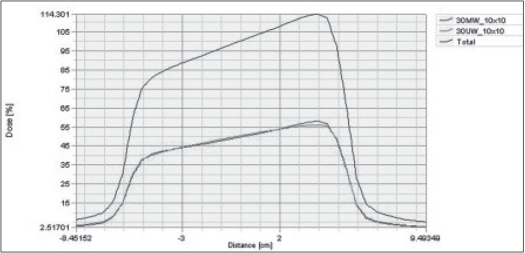
Shows the comparison of calculated dose profile for MW and UW for 30° at 10 cm depth 10 × 10 cm^2^ field size.

From homogeneous phantom generated in TPS, the calculated CADD for 10 × 10 cm^2^ field size of motorized and universal 60°-wedge was in good agreement. Figure 2 shows the comparison of calculated CADD of MW and UW for 30° at 10 cm depth for 10 × 10 cm^2^ field size. Figure 3 shows the comparison of calculated dose profile for MW and UW for 30° at 10 cm depth 10 × 10 cm^2^ field size.

The variation of 1, 1.5, 1.4 and 2% was found between measured doses by ion chamber and planned (calculated) dose at 10 cm depth for MW 15°, 30°, 45° and 60° at 10 cm depth respectively. The results are illustrated in Table 2. The planned dose was 2 Gy at 10 cm depth for 60° MW for 10 × 10 cm^2^ field size.

**Table 2:** Absolute dose verification in machine for motorized wedge 15°, 30°, 45° and 60° at 10 cm depth for 10 × 10 cm^2^ field size

*MW (degrees)*	*Measured dose (Gy)*	*Planned dose (Gy)*	*% variation*
15	1.98	2	1
30	1.97	2	1.5
45	1.972	2	1.4
60	1.96	2	2

Table 3 shows the comparison of treatment time calculated by TPS and calculated manually. Maximum variation of 0.59% was observed in the manual calculation and the TPS calculation.

**Table 3:** Comparison of treatment time calculated by treatment planning system for various field sizes with MW60° wedge at 10 cm depth and the manual calculation

*Field size (cm^2^)*	*Treatment time (minutes)*		
	
	*MW60*	*Manual*	*% variation*
5	5.02	5.05	0.59
10	4.37	4.37	Nil
15	4.03	4.01	0.5
15 × 20	3.92	3.92	Nil

Table 4 shows the weight factor for MW 15°, 30°, 45° and 60° for 10 × 10 cm^2^ field size. By using these appropriate beam weights, it is possible to generate isodose distributions. which exhibit effective wedge angles ranging from 0 to 60°. These weight factors were achieved by tilting the isodoses at 10 cm depth in a homogeneous phantom for a desired MW angle. These weight factors can be used for clinical implementation of MW during treatment planning.

**Table 4:** Weight factors for motorized wedge 15°, 30°, 45° and 60° for clinical implementation. These factors were calculated by tilting the isodoses for desired wedge angle

*Eclipse calculated wedge angle (degrees)*	*Wedged beam weight factor*	*Open beam weight factor*
15	0.16	0.84
30	0.38	0.62
45	0.63	0.37
60	1	1

The measured and calculated MW angles were in well agreement within + 2°. Our results were in well agreement with the requirements reported in the literature.[5–7] Table 5 shows the comparison of calculated and measured motorized wedge angle for 10 × 10 cm^2^ field size at 10 cm depth. The MW angle measured by CA24 was found to be 28.5° while the calculated MW angle was 30°. The wedge angles were measured from the isodoses generated from dose profiles measured at 4 depths with chamber array (CA24).

**Table 5:** Comparison of calculated and measured motorized wedge angle for 10 × 10 cm^2^ field size at 10 cm depth. The wedge angles were measured from the isodoses generated from dose profiles measured at 4 depth with chamber array (CA24). The measured and calculated motorized wedge angles were in well agreement within 2 degrees

*Calculated wedge angle (degrees)*	*Measured wedge angle (degrees)*	*Difference in measured and calculated wedge angle (degrees)*
15	13.4	-1.6
30	28.5	-1.5
45	46.3	1.3
60	59.5	-0.5

## Discussion

This study was an attempt to describe configuration of unique and novel treatment device, Theratron Equinox-80 cobalt unit, which is definitely of interest to the Medical Physics community from the developing countries. Usually the telecobalt units are available with symmetric collimation and individualized wedges. Till now, the concept of advance technology with the wedges was used in linear accelerators only. Installation of a modern telecobalt unit at our center provided the opportunity to investigate the optimal clinical implementation of the MW filter. This is the first machine in the world and was first installed at our center. The comparative data for MW is not available in the literature as on today.

Our measured data of CADD and profiles for MW were compared with the data supplied by the manufacturer generated with the same water phantom. The data sheets were also supplied by the manufacturer for MW 60° only and our results were in well agreement with their data as shown in Table 1. The data sheets were also provided by the manufacturer for four UW (15°, 30°, 45° and 60°) for comparison.

Calculated CADD and profiles for MW and UW were compared for 15°, 30°, 45° and 60°. Our results from Figure 2 show the comparison of CADD for UW and MW. The CADD were compared for 10 × 10 cm^2^ for wedge angles of 15°, 30°, 45° and 60°. Similarly Figure 3 shows the comparison of calculated and measured profile of UW (physical wedge) and MW. Thus the results were acceptable for clinical implementation for these 4 MW angles.

It is understood that the treatment time in TCU is mentioned in minutes. It is to be noticed that eclipse gives the treatment time for Cobalt machine in MU but it is in seconds, which then can be converted to minutes. We have verified the TPS treatment time calculation of MW with the manual calculation for other MW angles (15°, 30° and 45°) also and the results were encouraging. Eclipse also takes care of the decay of the radionuclide into account and corrects the treatment time as well. The manual treatment time for Equinox-80 machine for a particular field was calculated by the equation (4) given below:

(4) Teatment Time (min)=Prescribed dose (Gy)/[Dose rate (Gy/min)×CADD×wedge factor]

Table 4 shows the weight factor for MW (15°, 30° and 45° and 60°) for 10 × 10 cm^2^ field size. The weight factors were checked for other field sizes (5, 15 and 15×20 cm^2^) also. We found that the variation in weight factor with field size was not significant from the clinical point of view and the same weight factor of 10 × 10 cm^2^ can be used for the particular MW angle. These weight factors can be used for clinical implementation and the system gives the treatment time for open and the MW for a particular beam weight.

CA24 was found very useful in measuring the MW angles compared to the single ion chamber. In MW treatment, the source movement is twice, i.e., source delivers open beam radiation and goes back. The MW comes in the radiation filed and again the source delivers the radiation. For such measurements, single ion chamber fails to measure the profile. CA24 has been used as a quality assurance tool in the MW angle verification.

## Conclusion

We have configured MW at four wedge angles (15°, 30° and 45° and 60°). The MW data for our new telecobalt Equinox-80 was successfully configured in eclipse TPS. The MW provides the capability of modifying the isodose characteristics of the radiation beam same as the universal wedges and can safely be used for clinical applications. However, to utilize the clinical advantages of MW, accurate dose calculation and constraints checking are required during the treatment planning process.
